# StAR Protein Stability in Y1 and Kin-8 Mouse Adrenocortical Cells

**DOI:** 10.3390/biology4010200

**Published:** 2015-03-04

**Authors:** Barbara J. Clark, Elizabeth A. Hudson

**Affiliations:** Department of Biochemistry and Molecular Biology, Center for Genetics and Molecular Medicine, University of Louisville School of Medicine, Louisville, KY 40292, USA; E-Mail: eahunt02@exchange.louisville.edu

**Keywords:** STAR, phosphorylation, protein stability, proteasome

## Abstract

The steroidogenic acute regulatory protein (STAR) protein expression is required for cholesterol transport into mitochondria to initiate steroidogenesis in the adrenal and gonads. STAR is synthesized as a 37 kDa precursor protein which is targeted to the mitochondria and imported and processed to an intra-mitochondrial 30 kDa protein. Tropic hormone stimulation of the cAMP-dependent protein kinase A (PKA) signaling pathway is the major contributor to the transcriptional and post-transcriptional regulation of STAR synthesis. Many studies have focused on the mechanisms of cAMP-PKA mediated control of STAR synthesis while there are few reports on STAR degradation pathways. The objective of this study was to determine the effect of cAMP-PKA-dependent signaling on STAR protein stability. We have used the cAMP-PKA responsive Y1 mouse adrenocortical cells and the PKA-deficient Kin-8 cells to measure STAR phosphorylation and protein half-life. Western blot analysis and standard radiolabeled pulse-chase experiments were used to determine STAR phosphorylation status and protein half-life, respectively. Our data demonstrate that PKA-dependent STAR phosphorylation does not contribute to 30 kDa STAR protein stability in the mitochondria. We further show that inhibition of the 26S proteasome does not block precursor STAR phosphorylation or steroid production in Y1 cells. These data suggest STAR can maintain function and promote steroidogenesis under conditions of proteasome inhibition.

## 1. Introduction

Trophic hormone activation of the cAMP-dependent protein kinase A (PKA) signaling pathway is the major mediator of steroidogenesis in the adrenal and gonads. The cAMP-PKA pathway activates cholesterol delivery to the mitochondrial inner membrane for the first step in steroid biosynthesis and increases transcriptional activation of genes encoding steroidogenic enzymes (reviewed in [1]). The transport of cholesterol into mitochondria is the rate-limiting step in steroidogenesis and this step is dependent upon the expression and function of the steroidogenic acute regulatory protein (STAR, encoded by the STARD1 gene) [2,3]. STAR is a nuclear-encoded gene that contains an amino-terminal mitochondrial signal sequence that targets the protein to the mitochondria where it is imported, with cleavage of the signal sequence, resulting in the intra-mitochondrial 30 kDa form of STAR (reviewed in [4]). The processed 30 kDa STAR localized to the mitochondria matrix is not active in cholesterol transport [5,6]. Rather, STAR association with the outer mitochondrial membrane is required for its activity. Models of STAR-mediated cholesterol transport mechanisms have been reviewed and implicit in these models is the requirement for continued synthesis of precursor STAR to sustain the transient interactions of the newly synthesized protein with the mitochondria outer membrane (reviewed in [7,8,9]).

cAMP-PKA-dependent mechanisms contribute to the transcriptional and post-transcriptional regulation of STAR expression and activity (reviewed in [10]). It is well accepted that STAR activity is enhanced by PKA-dependent phosphorylation of serine 194/195 (murine/human) on precursor STAR [11,12,13,14,15] Furthermore, STAR mRNA localization to mitochondria and interaction with mitochondrial A-kinase anchoring protein 121 (AKAP121) has been proposed as a mechanism to co-localize STAR translation with type II PKA to enhance localized translation and phosphorylation of STAR at the mitochondria [16,17,18]. To date only a causal link between STAR phosphorylation and function is established and the mechanism for enhanced activity of phosphorylated STAR remains to be determined.

The mitochondrial 30 kDa STAR retains the phosphorylation state but there is no known function for STAR within the mitochondria. Mechanisms for degradation of 30 kDa STAR are proposed to protect the mitochondria from accumulation of a non-functional protein [19,20]. On the other hand, the precursor STAR is very labile with half-life estimates of ≤5 min that were based on rates of processing the signal sequence [21,22]. Precursor STAR processing to the mitochondrial 30 kDa form is reported to be very efficient and stoichiometric [21], suggesting that the newly synthesized precursor STAR is not degraded in the cytosol under normal conditions. However, disruption of the mitochondrial membrane potential to block protein import does not result in accumulation of the STAR precursor, indicating that in the absence of mitochondria import; e.g., under aberrant conditions, STAR is rapidly degraded in the cytosol [23,24]. Previously it was reported that inhibition of the 26S proteasome resulted in accumulation of precursor STAR and it is proposed that STAR is a substrate for the 26S proteasome [19,25]. What has not been addressed is whether STAR phosphorylation contributes to STAR protein stability.

In the adrenal, adrenocorticotropic hormone (ACTH) activates the cAMP-PKA signal transduction pathway. The requirement for PKA activity in ACTH-stimulated steroidogenesis has been well defined using the Y1 mouse adrenocortical cell line and the non-responsive Kin mutant subclones [26]. The lack of ACTH response in the Kin subclones is due to point mutations in the regulatory subunits of PKA that decrease cAMP binding and enzyme activity. The Kin-8 cell line is the least responsive to ACTH or cAMP [27]. Kin-8 cells have low to undetectable levels of the steroid hydroxylase genes; e.g., CYP21, CYP11A (P450scc), and the expression of these genes is not increased after hormonal stimulation [28]. We previously reported that the Kin-8 cells express 50% of the STAR mRNA and protein compared to Y1 cells and that 8Br-cAMP-treatment resulted in a similar fold-increase in STAR protein synthesis in both cell lines [29]. The current study has taken advantage of STAR expression in the Kin-8 cells to address whether cAMP-PKA signaling affects STAR protein stability.

## 2. Experimental Section

### 2.1. Source of Reagents

Nutrient Mixture F-10 Ham, 8-bromoadenosine 3':5'-cyclic monophosphate (8-Br-cAMP), Adrenocorticotropin hormone, fragment 1–14 (ACTH), MG132), L-Methionine, and L-Cysteine were all purchased from Sigma-Aldrich, Inc. Fetal Bovine Serum, Horse Serum, penicillin/streptomycin, and L-Glutamine were obtained from Invitrogen Life Sciences (Carlsbad, CA, USA). Epoxomicin was supplied by Calbiochem (EMD Chemicals, Gibbstown, NJ, USA). RPMI media was from Mediatech, Inc. (Manassas, VA, USA) and Expre^35^S^35^S [^35^S]Protein Labeling Mix was purchased from Perkin Elmer Life Sciences.

### 2.2. Radiolabeling Studies and STAR Immunoprecipitation

Y-1 and Kin-8 cells were maintained in F-10 Ham medium containing 10% horse serum, 2.5% fetal bovine serum, and 0.5% antibiotic/antimycotic. The cells were plated in 60-mm^2^ tissue culture dishes at 2 × 10^6^ cells/dish two days prior to treatment. To radiolabel the cellular protein, cells were washed twice with PBS and pretreated for 40 min in serum-free RPMI media without L-methionine, or L-cysteine. Cells were then incubated for 2 or 6 h with fresh serum-free RPMI media containing 1.5 µg/mL L-methionine and 0.4 mCi/mL ^35^S-methionine. In some cases the cells were treated with either 1 mM 8-Br-cAMP in the absence or presence of either 10 µM MG132 or 10 µM epoxomicin during the labeling period as indicated. To determine 30 kDa STAR protein half-life (t½) in Y1 and Kin-8 cells, the cells were radiolabeled for 2 h in the presence of 8Br-cAMP, washed, then placed in fresh RPMI media (no 8Br-cAMP) containing 15 µg/mL L-methionine and cell lysates were collected after 0, 30, 60, 90, 120, or 240 min chase times. To determine STAR precursor protein t½ in Y-1 cells and the effect of cAMP on STAR t½, the cells were radiolabeled for 6 h in the presence of 150 nM ACTH + 10 µM epoxomicin followed by a cold chase in fresh media and in either the absence or presence of 150 nM ACTH for 0, 15, 30, 60, 90, 120, or 240 min. At the end of treatment or chase times, cell lysates were prepared and STAR immunoprecipitated as previously described [29,30]. In brief, STAR was immunoprecipitated from lysates using equivalent TCA-precipitable counts and recovered using protein A agarose beads. As a positive control for STAR immunoprecipitation, MA-10 mouse Leydig tumor cells were radiolabeled with [^35^S]-methionine for 2 h in the presence of 8Br-cAMP and STAR processed as above in parallel with the Y1 and Kin 8 cells. Equal volumes of protein samples were separated on 12.5% SDS-PAGE and the gel was fixed in 40% methanol/10% acetic acid, processed with Entensify^®^ Universal Autoradiography Enhancer (Perkin Elmer), dried, and exposed to a phosphorscreen followed by autoradiography. The data were analyzed using ImageQuant (Version 5.1, Molecular Dynamics) or Un-Scan-It (Silk Scientific) software. The t½ for STAR was determined for each independent experiment and the reported half-life values are the mean ± SEM from all experiments.

### 2.3. Immunoblot Analysis

Y1 and Kin-8 cells were plated as above and the cells were treated for 2 and 6 h in serum free medium in the presence or absence of 1 mM 8-Br-cAMP ± 10 µM MG132 or 10 µM epoxomicin. Media and cell pellets were collected and progesterone levels and STAR protein levels measured by radioimmunoassay and Western blot, respectively, as described below. Mitochondria or whole cell lysates were harvested in 10 mM Tris/Cl pH 7.4, 0.25 M sucrose, 0.1 M EDTA, 1 mM PMSF, 0.4 µ/mL aprotinin, and 1 µg/mL leupeptin. Equal amounts of protein, as determined by Bradford assay, were separated on 12.5% SDS-PAGE and transferred to PVDF membrane. Standard western blot procedures were followed as previously described using either purified rabbit α-phospho-STAR (pSTAR; generated against a peptide conjugated to keyhole limpet hemocyanin corresponding to amino acids 190–199 of mouse STAR with Ser194 phosphorylated [31]) or purified rabbit α-GST-STAR IgG for total STAR (tSTAR) followed by an HRP-conjugated donkey anti-rabbit secondary antibody [29,30]. The membranes were first probed for phosphoSTAR (pSTAR), then stripped and reprobed for total STAR (tSTAR). Following tSTAR immunoblot, the whole cell lysate samples were probed for β-actin (β-actin Rabbit mAb-HRP Conjugate #5125, Cell Signaling Technol.) and the mitochondria samples were probed for Tom40 (Tom40(H-300), Santa Cruz Biotech.) as loading controls. Immunospecific bands were detected using the Western Lightning Chemiluminescent Kit (Amersham) and quantified using Un-Scan-It software (Silk Scientific Corp.) with scanned gel images (ScanWizard Pro., Microtek Labs, Inc., Carson, CA, USA). Multiple exposure times and multiple protein concentrations were used to define the linearity of the response.

### 2.4. Progesterone Radioimmunoassay

Progesterone levels in collected medium were determined by incubation with [^3^H]Progesterone (Amersham Biosciences) and progesterone antibody (Sigma-Aldrich). Unbound progesterone was removed by charcoal extraction, and bound progesterone was measured by scintillation counting. The progesterone levels were determined by extrapolation from a standard curve. The mean values for ng steroid/mL medium (±S.D.) were determined, and the data normalized to the control level, which was set to 1.0.

### 2.5. Statistical Analysis

A statistical significant difference for either STAR protein expression or progesterone levels between inhibitor treatment and control were determined using an unpaired Student’s *t*-test. pSTAR/tSTAR IOD ratios in Epox- and MG132-treated cells were compared using a Mann-Whitney statistical analysis. *p* ≤ 0.05 was considered statistically significant (GraphPad Software, San Diego, CA, USA).

## 3. Results

### 3.1. STAR Phosphorylation and Protein Half-Life in Y1 and Kin-8 Cells

**Figure 1:**
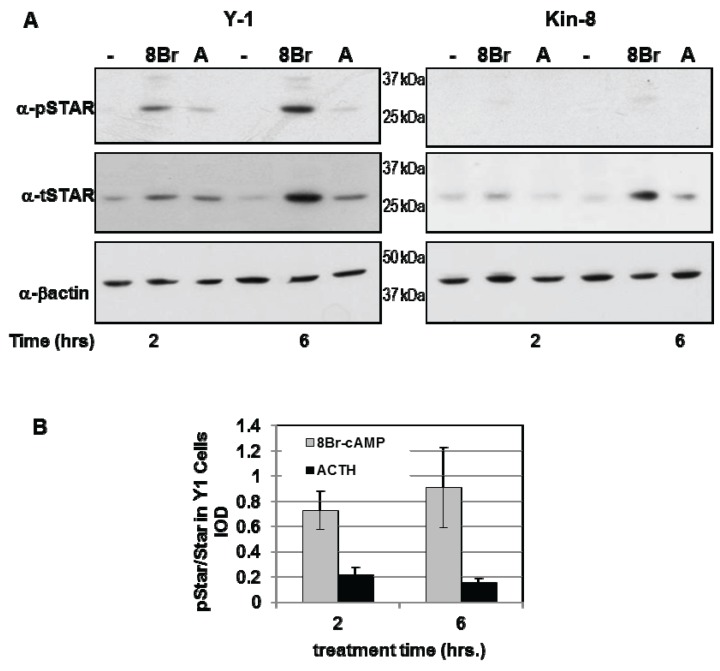
PhosphoSTAR protein expression in Y1 cells. Y1 and Kin-8 cells were placed in serum-free medium overnight then treated for 2 and 6 h in serum-free medium in the absence (−) or presence of either 1 mM 8Br-cAMP or 150 nM adrenocorticotropic hormone (ACTH) as indicated. Western blot analysis was used to detect phosphoSTAR followed by total STAR protein levels in 15 µg of whole cell lysate as described in the Experimental Section. (**A**) Shown is a representative Western blot for phospho-STAR (α-pSTAR) and total STAR (α-tSTAR) from three independent experiments; (**B**) The IOD for pSTAR and tSTAR were determined as detailed in the Experimental Section and the pSTAR/tSTAR ratio in Y1 cells after 2 and 6 h treatment was determined for each individual experiment. Shown are the mean values ± SEM (*n* = 3).

Y1 and Kin-8 cells were treated with either 8Br-cAMP or ACTH for 2 h and 6 h and the levels of phosphoSTAR (pSTAR) and total STAR (tSTAR) were determined by Western blot analysis (Figure 1A). Consistent with our previous study, STAR expression in Kin-8 cells was detectable after 8Br-cAMP treatment with protein levels 50% of that detected in Y1 cells [29]. Phosphorylated STAR protein was undetectable in untreated Y1 and Kin-8 cells (Figure 1A) and 8Br-cAMP or ACTH treatment resulted in the appearance of pSTAR protein in only in the Y1 cells. The pSTAR/tSTAR ratio was 4-fold greater after8Br-cAMP treatment compared to ACTH treatment (Figure 1B). These data demonstrate for the first time that newly synthesized STAR is not phosphorylated in Kin-8 cells.

**Figure 2:**
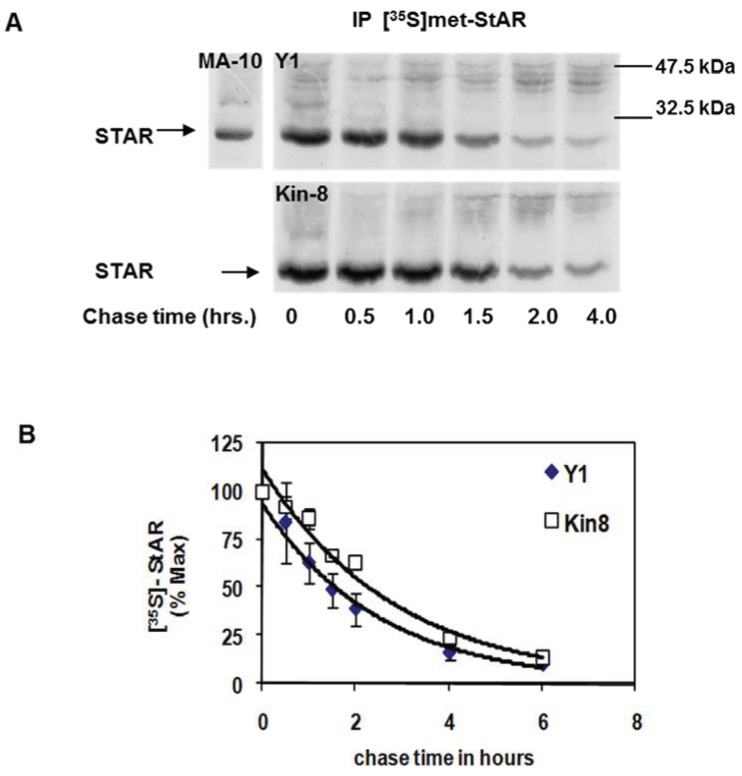
Mitochondrial STAR protein half-life in Y1 *vs.* Kin-8 cells. Y1 or Kin-8 cells were radiolabeled with [^35^S]-methionine for 2 h in the presence of 8Br-cAMP followed by cold chase in the absence of 8Br-cAMP for the indicated times. (**A**) STAR was immunoprecipitated from cell lysates as described in the Experimental Section and shown is a representative fluorography from five or three independent experiments for Y1 and Kin-8, respectively. MA-10, STAR immunoprecipitated from radiolabeled MA-10 mouse Leydig tumor cell lysates (**B**) The integrated optical densities (IOD) for STAR was determined as detailed in Experimental Section and the values were expressed as percent of *t* = 0 chase time that was set to 100%. Shown are the mean relative percent values ± SEM (*n* = 5, Y1; *n* = 3 Kin-8). The t_1/2_ was determined for each experiment independently and the mean value for Y1 was significantly different compared to Kin-8 based on a Student’s *t*-test with *p* < 0.05.

To assess whether cAMP-PKA signaling contributes to STAR protein stability, we compared the half-life of STAR between the Y1 and Kin-8 cell lines. Using a standard pulse-chase radiolabeling approach, the cells were treated with 8Br-cAMP for 2 h in the presence of [^35^S]-methionine followed by a cold chase for 4 h in the absence of 8Br-cAMP. STAR protein was recovered by immunoprecipitation and radiolabeled STAR detected by fluorography (Figure 2A). STAR protein half-life in Y1 and Kin-8 cells was determined to be 1.5 ± 0.11 h and 2.2 ± 0.06 h, respectively (*p* < 0.05). Thus, the half-life of 30 kDa STAR is 30% longer in the Kin-8 cells compared to the Y1 cells.

**Figure 3:**
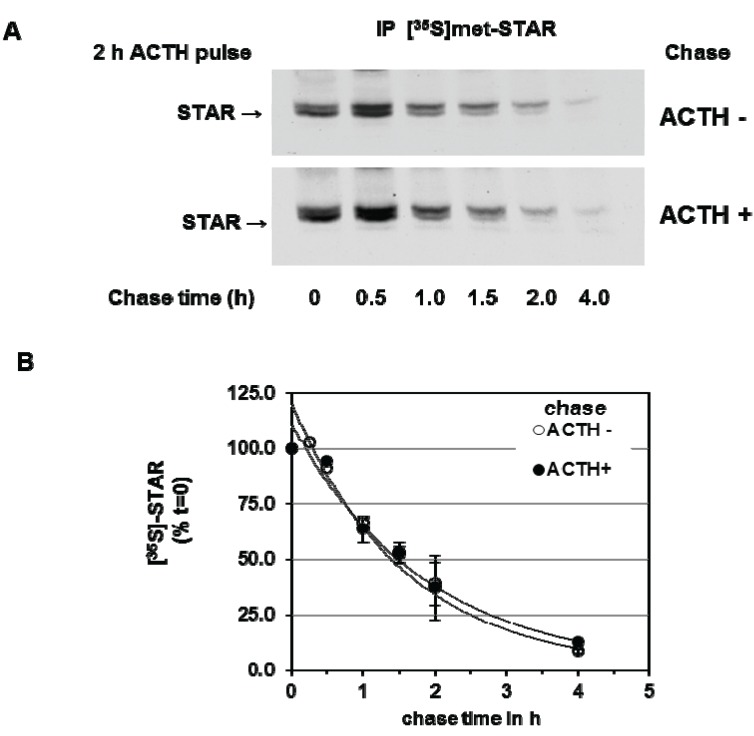
cAMP-PKA activation and mitochondrial STAR protein half-life in Y1 cells. Y1 cells were radiolabeled with [^35^S]-methionine for 6 h in the presence of 150 mM ACTH followed by cold chase in either the absence or presence of ACTH for the indicated times. (**A**) STAR was immunoprecipitated from cell lysates as described in the Experimental Section and shown is a representative fluorography from three independent experiments; (**B**) The IOD for STAR was determined and the values were expressed as percent of *t* = 0 chase time that was set to 100%. Shown are the mean relative percent values ± SEM (*n* = 3).

We next determined the effect of PKA activation during the chase period on 30 kDa STAR half-life. Y1 cells were stimulated with ACTH for 2 h in the presence of [^35^S]-methionine followed by a cold chase for 4 h in the absence or presence of ACTH. As shown in Figure 3, addition of ACTH during the chase period did not affect STAR half-life, the t_1/2_ was determined to be 1.4 ± 0.13 h in the presence of ACTH compared to 1.4 ± 0.05 h in the absence of ACTH. Together the data indicate that the degradation of STAR 30 kDa protein in Y1 cells is not regulated by a PKA-dependent mechanism.

### 3.2. Precursor STAR Expression in Y1 Cells

**Figure 4:**
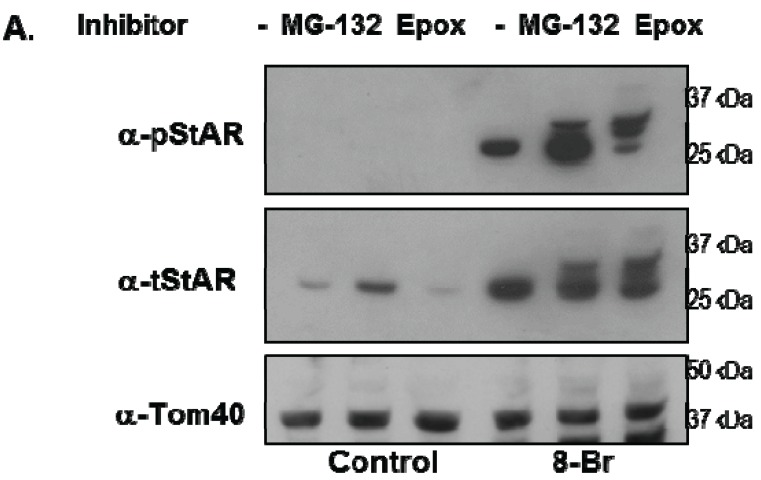

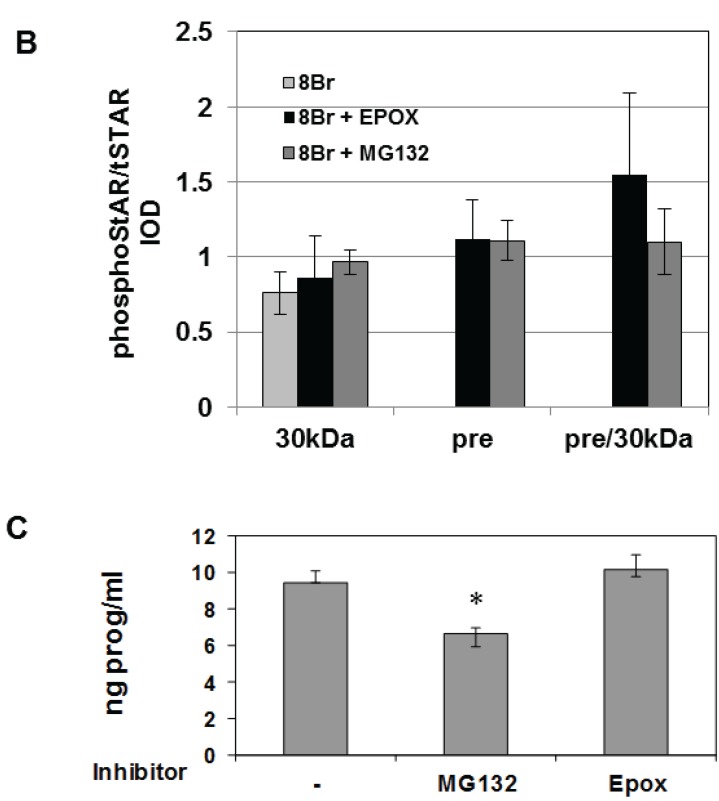
The effect of proteasome inhibitors on STAR phosphorylation in Y1 cells. Y1 cells were treated in serum -free medium in either the absence (Control) or presence of 1 mM 8Br-cAMP and 10 µM MG132 or 10 µM epoxomicin (Epox) as indicated. After 6 h the media was collected for progesterone assays and mitochondria were isolated from cell pellets. (**A**) PhosphoSTAR (pSTAR) was detected by standard Western blot then the membrane was stripped and reprobed for total STAR (tSTAR). Shown is a Western blot for pSTAR and tSTAR expression in 10 μg of isolated mitochondria. α-Tom40 was used as a loading control. Similar results were obtained in two additional experiments. (**B**) The integrated optical densities (IOD) for phosphoSTAR and total STAR for each STAR immunoreactive band (precursors and 30 kDa) were measured and the pSTAR/tSTAR IOD ratio was determined for the 8Br-cAMP-treated samples. Pre/30 kDa is the precursor/30 kDa pSTAR/tSTAR values. Shown are the mean values ± SEM from three independent experiments. There is no significant difference between the Epox and MG-132 treatments on pre/30 kDa pSTAR/tSTAR IOD based on a Mann-Whitney statistical analysis. (**C**) Progesterone levels were measured in the cell medium and shown are the mean values for ng progesterone/mL ± SD from 3 independent experiments for the 8Br-cAMP-treated samples in the absence (−) and presence of proteasome inhibitors. * indicates a significant decrease in progesterone in the MG132 treated sample relative 8Br-cAMP alone (−), *p* < 0.05 based on a Student’s *t*-test.

Y1 cells were treated with 8Br-cAMP either in the absence or presence of the 26S proteasome inhibitors epoxomicin (Epox) or MG132 and phosphoSTAR (pSTAR) and total STAR (tSTAR) protein were detected by Western blot analysis of isolated mitochondria. Addition of either MG132 or Epox resulted in accumulation of precursor STAR in the 8Br-cAMP-treated cells (Figure 4A, tSTAR panel). Probing with the phospho-specific STAR antibody, we confirm that 8Br-cAMP treatment alone increased 30 kDa STAR protein expression and pSTAR (Figure 1A and Figure 4A). In addition, we now demonstrate that precursor STAR is phosphorylated in the presence of proteasome inhibitors (Figure 4A, pSTAR panel). The pSTAR: tSTAR ratio for 30 kDa STAR was similar for all treatment groups, suggesting inhibition of the proteasome did not significantly disrupt STAR phosphorylation or mitochondria import (Figure 4B). The pSTAR: tSTAR ratio for precursor STAR was similar for MG132 and Epox-treated cells, although the precursor pSTAR relative to 30 kDa pSTAR for Epox- treated cells trended toward an increase compared to the MG132 treated cells (pre/30 kDa, Figure 4B). In addition, MG132 treatment resulted in an increase in 30 kDa STAR expression, which is most apparent in the control cells that were not treated with 8Br-cAMP (Figure 4A). To determine whether proteasome inhibition affected STAR function, progesterone levels in the cell medium were measured after treatment of Y1 cells with 8Br-cAMP in the absence of presence of MG132 or Epox. As shown in Figure 4C, Epox treatment had no effect on 8Br-cAMP-stimulated progesterone production while MG132 treatment resulted in a 30% decrease in steroid output.

**Figure 5:**
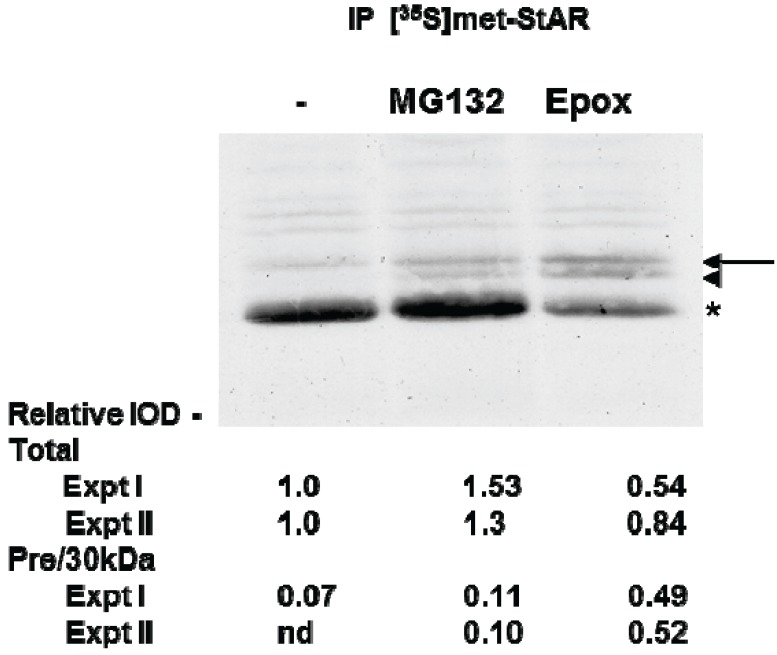
The effect of proteasome inhibitors on STAR synthesis in Y1 cells. Y1 cells were radiolabeled with [^35^S]-methionine for 2 h in the presence of 1 mM 8Br-cAMP alone (−) or 8Br-cAMP plus either 10 µM MG132 or 10 µM epoxomicin (Epox) as indicated. STAR was immunoprecipitated from cell lysates as described in the Experimental Section and shown is a fluorograph representative of two independent experiments. The IOD for each of the STAR bands (precursor, denoted by arrow and arrowhead and 30 kDa, denoted by the asterisk) was quantitated independently and either summed to determine the Total relative expression or the precursor bands were summed to determine the ratio of precursor to 30 kDa expression (Pre/30 kDa). The IOD values were normalized to the 8Br-cAMP sample (−) that was set to a value of 1.0 and shown are the results from two independent experiments.

To directly determine whether proteasome inhibition affected STAR synthesis and 30 kDa STAR expression, we used [^35^S]-methionine radiolabeling of Y1 cell proteins followed by immunoprecipitation of tSTAR. As expected, radiolabeled STAR increased with 8Br-cAMP treatment and precursor STAR was detected only in the presence of either MG132 or Epox (Figure 5). Using this assay we determined total STAR levels (precursor + 30 kDa form) were decreased 20%–50% in the presence of Epox with the precursor protein forms representing 50% of the total STAR expression. In contrast, MG132 treatment resulted in a 30% increase in 30 kDa STAR expression, shifting the distribution towards 30 kDa STAR relative to the precursor protein (Figure 5). Together the data support proteasome inhibition does not affect STAR phosphorylation. However, Epox and MG132 treatments have differential effects on both STAR synthesis and activity.

### 3.3. Precursor STAR Stability in Epox-Treated Y1 Cells

**Figure 6:**
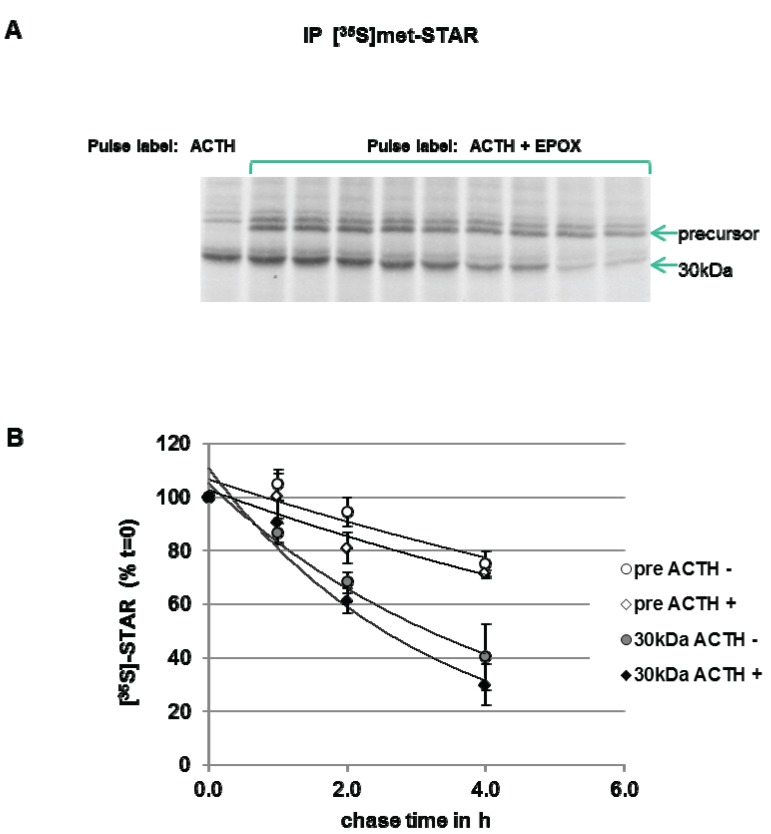
Effect of ACTH and proteasome inhibition on STAR precursor half-life in Y-1 cells. Y1 cells were treated with 150 nM ACTH in the absence (−) or presence (+) of 10 µM epoxomicin (Epox) and pulse radiolabeled with [^35^S]-methionine for 6 h. The cells were then subjected to a cold chase for the indicated times either in the absence (−) or presence (+) of 150 nM ACTH. STAR was immunoprecipitated from cell lysates as described in the Experimental Section. (**A**) Shown is a fluorograph representative of three independent experiments. (**B**) The IOD for precursor STAR and 30 kDa STAR were determined and the values were expressed as percent of *t* = 0 chase time that was set to 100% to determine STAR half-life (t_1/2_) as detailed in the Experimental Section. Shown are the mean relative percent values ± SEM from three independent experiments plotted against chase time.

To measure STAR precursor protein half-life in Y1 cells, the cells were pulse-labeled with [^35^S]-methionine in the presence of ACTH plus Epox to accumulate STAR precursor proteins. After a 6 h pulse period, a cold chase in the absence of Epox was performed. As shown in Figure 6, precursor STAR is detected throughout the 4 h chase period, therefore, a half-life could not be determined. The 30 kDa STAR half-life was determined to be 2.5 ± 0.5 h. The half-life of 30 kDa STAR in ACTH-treated cells in the absence of Epox is 1.4 ± 0.13 h (Figure 3), indicating that Epox treatment increased STAR 30 kDa half-life. Addition of ACTH to maintain PKA activation during the chase did not affect precursor STAR or 30 kDa STAR protein levels, supporting no effect of PKA activation on STAR degradation rates (Figure 6). These data indicate that long term (6 h) Epox-mediated proteasome inhibition stabilizes both precursor and 30 kDa STAR protein.

## 4. Discussion

We have utilized the Y1 and Kin-8 mouse adrenocortical cell lines to demonstrate that the PKA-deficient Kin-8 cells synthesize the STAR protein in response to 8Br-cAMP, but do not effectively phosphorylate STAR. Therefore, these cell culture systems were used to test whether PKA-dependent STAR phosphorylation was correlated with protein stability. We show that the half-life of 30 kDa STAR is modestly increased in the PKA-deficient Kin-8 cells compared to the Y1 cells. One interpretation is these data is that phosphorylated 30 kDa STAR is degraded more rapidly in the mitochondria. Alternatively, the difference in 30 kDa STAR half-life may be due to cell-specific differences in mitochondrial protease activities. Recent studies have shown that STAR accumulation in the mitochondria triggers retrograde signaling that increases nuclear gene expression of the proteases responsible for its own degradation in the mitochondria [32]. We have shown that 8Br-cAMP treated Kin-8 cells express less 30 kDa STAR compared to Y1 cells ([29] and herein), therefore it is possible this retrograde signaling in Kin-8 cells is delayed or attenuated. In support of cell-specific differences contributing to modest difference in STAR half-life in Kin-8 cells, we show there is no difference in 30 kDa STAR half-life in Y1 cells treated with either ACTH or 8-Br-cAMP. Comparison of pSTAR: tSTAR ratio in ACTH-stimulated and 8Br-cAMP-stimulated Y1 cells indicates STAR phosphorylation is 4-fold lower in the ACTH-treated cells. Thus the level of phosphoSTAR is not correlated with protein half-life in Y1 cells.

Previous studies showed inhibition of the 26S proteasome resulted in precursor STAR accumulation in FSH-treated primary rat luteinizing granulosa cells [19] and forskolin-treated primary rat pre-ovulatory granulosa cells [25]. We show similar results in Y1 cells providing further support for cytoplasmic degradation of precursor STAR by the proteasome. We have extended this observation to show the pSTAR: tSTAR ratio for 30 kDa STAR is not affected by inhibition of the 26S proteasome. These data indicate that mitochondria import of pSTAR was not disrupted by blocking precursor STAR degradation. Thus proteasome inhibition did not lead to mitochondrial dysfunction as measured by active STAR phosphorylation and import, and progesterone synthesis.

Our observation that Epox treatment reduced 8Br-cAMP-stimulated STAR synthesis yet maintained progesterone synthesis can be explained by the presence of stable precursor STAR in the phosphorylated state. This explanation is consistent with the working model for STAR function where the active form is the phosphorylated precursor STAR localized to the mitochondria outer membrane. Indeed, the precursor: 30 kDa ratio for phosphorylated STAR was greater in Epox-treated cells supporting that the precursor STAR remained active and promoted steroid production [11,12,16,22,31,33]. Together our data show that blocking cytoplasmic precursor STAR degradation by inhibition of the 26S proteasome does not affect the PKA-dependent phosphorylation of precursor STAR or STAR function. It remains to be determined whether the degradation of a functional precursor STAR may represent a regulatory point to “turn off” STAR function under conditions of mitochondria dysfunction.

The modest increase in the steady-state levels of the 30 kDa STAR in MG132 treated Y1 cells may also reflect inhibition of STAR degradation in the mitochondria. MG132 was previously shown to inhibit mitochondrial proteases and delay 30 kDa STAR degradation [19,20]. The mechanism for degradation of the 30 kDa STAR in the mitochondria is proposed to occur in two phases: the first phase is mediated by an ATP-dependent Lon protease that is sensitive to MG132 inhibition while the second phase of degradation proceeds by a MG132-insensitive protease (reviewed in [20]). Thus, the increase in 30 kDa STAR in MG132 treated Y1 cells would be consistent with inhibition of the ATP-dependent Lon protease. We also measured a modest decrease in progesterone levels in the 8Br-cAMP + MG132 treated Y1 cells compared to 8Br-cAMP alone or 8Br-cAMP + Epox. Previous studies showed MG132 treatment of forskolin-treated primary rat pre-ovulatory granulosa cells resulted in increased precursor and 30 kDa STAR [25]. However, in that study MG132 treatment increased progesterone levels compared to our observed decrease in Y1 cells. It is possible that there may be cell-specific effects of MG132, such as other enzymes in the steroidogenic pathway or other targets not measured in this study are modestly affected in Y1 cells.

The pulse-chase experiment with inhibition of the 26S proteasome with Epox resulted in stabilization of both precursor and 30 kDa STAR. Epox is an irreversible inhibitor of the proteasome, therefore, proteasome inhibition was likely sustained during the chase period. Nevertheless, this was an unexpected finding as we anticipated that some of the precursor STAR that accumulated upon Epox treatment would be converted to 30 kDa STAR during the chase period. Therefore, we cannot exclude the possibility that over time the sustained inhibition of the proteasome may either suppress mitochondrial function for STAR import, or that the accumulated and stabilized precursor STAR is no longer competent for import. In addition, the prolonged half-life of the 30 kDa STAR in Epox-treated cells suggests additional effects of this inhibitor with longer treatment times. Previously 30 kDa STAR half-life in primary rat luteinizing granulosa cells was not affected when Epox was added only during the chase period [19], supporting that the longer treatment times use herein may have additional effects.

## 5. Conclusions

This study shows for the first time that PKA-dependent phosphorylation of STAR does not contribute to 30 kDa STAR protein stability. Although we observed a modest increase in 30 kDa STAR half-life in Kin-8 cells compared to Y1 cells, we propose this difference reflects differences between the cell types. This conclusion is supported by our observation that differences in STAR phosphorylation levels in Y1 cells did not affect protein stability.

The presence of phosphorylated precursor STAR and active steroid production under conditions of Epox-mediated proteasome inhibition indicates PKA-dependent control of STAR phosphorylation and function remains intact. This novel finding is consistent with the model for STAR precursor proteins sustaining steroidogenesis. We conclude that proteasome inhibition does not affect cAMP-PKA-dependent STAR function and that the PKA-dependent phosphorylation status of STAR does not contribute to STAR turn-over in the mitochondria.
